# Simulating potential yield of rainfed soybean in northeast Nigeria

**DOI:** 10.1016/j.eja.2022.126683

**Published:** 2023-01

**Authors:** Alpha Y. Kamara, Jenneh F. Bebeley, Kamaluddin T. Aliyu, Abdullahi I. Tofa, Lucky Omoigui, Reuben Solomon, Folorunsho M. Akinseye

**Affiliations:** a International Institute of Tropical Agriculture, Sabo Bakin Zuwo Rd, Kano, Nigeria; b Sierra Leone Agricultural Research Institute, Freetown, Sierra Leone; c International Crops Research Institute for the Semi-Arid Tropics (ICRISAT) Kano, Nigeria; d Centre d′étude régional pour l′amélioration de l′adaptation à la sécheresse (CERAAS), Thies Escale, Senegal

**Keywords:** CROPGRO-Soybean model, Optimum sowing window, Soybean, Productivity, Northeast Nigeria

## Abstract

We used the CROPGRO-Soybean model to simulate the production potential of rainfed soybean in northeast Nigeria. Data from ten soybean experiments conducted under optimal conditions in 2016–2018 at Kano and Dambatta in the Sudan savanna (SS) agroecological zone were used to determine the cultivar coefficients and calibrate the model for the varieties TGX 1448–2E and TGX1951–3 F. The model was evaluated with data from four phosphorous response trials conducted at Zaria and Doguwa in the northern Guinea savanna (GS) of Nigeria between 2016 and 2018. Results show that the CROPGRO-Soybean model was able to accurately simulate soybean growth and grain yield with low RMSE and high d-index values. Consequently, the model was used to investigate the rainfed yield potential of the two varieties in 24 sites in northeast Nigeria under different sowing windows using 30-year (1985–2014) weather data. The result shows that soybean can be grown in northeast Nigeria, but yield performance is dependent on location, variety and sowing window. The simulated yield was higher in the SS than in the GS agro-ecozone despite the longer growing period in the later. Low yield was simulated for TGX 1448–2E for most of the sites. The yield of TGX1951–3 F was above a threshold of 1500 kg ha^−1^ in 5 out of 12 sites in the GS and 7 out of 12 sites in the SS, suggesting that this variety is the most suitable for cultivation in northeast Nigeria. Sowing TGX 1951–3 F can be delayed to July 16 at Gwaskara, Nasarawo Demsa and Tawa in the GS and at Briyel, Lakundum, Jara Dali, Kurbo Gayi, and Mathau in the SS with a low-risk of crop failure. The desired yield will be achieved at Chikala and Puba Vidau with a significantly low risk of crop failure for all sowing windows. The results from this study suggest that the CSM-CROPGRO-Soybean model can be a valuable tool in determining the right variety and sowing window for soybean production in targeted agroecological zones in northeast Nigeria.

## Highlights

• The CROPGRO-Soybean model was calibrated and validated for two varieties of soybean TGX1951–3 F and TGX1448-2E. The model accurately reproduced the observed values.
• A 30-year seasonal analysis showed that the simulated grain yield was generally higher in the Sudan savanna (SS) than in the Guinea savanna (GS) zone.
• Higher yields were simulated for TGX1951–3 F than for TGX 1448-2E across planting windows and sites.
• Best sowing window depends on location and variety, but TGX1951–3 F generally has a wider sowing window than TGX1448-2E

## 1 Introduction

Among the grain legume crops, soybean is an important cash crop for rural households in the Nigerian savannas and other parts of sub-Saharan Africa (SSA) partly due to demand for processing at an industrial scale (Ugbabe et al., 2017). It is promoted among smallholders not only for food and cash but for improving soil fertility in cereal-dominated cropping systems through biological nitrogen fixation (Ulzen et al., 2018, Vanlauwe et al., 2019). When grown in rotation, it reduces the infestation of parasitic weeds in crop fields (Franke et al., 2004, Kamara et al., 2008). Because of high demand for animal feeds, soybean processing has expanded in Nigeria and has overtaken that of groundnut (Rusike et al., 2013) making soybean the most important oil crop in Nigeria. Because of the importance of soybean, several improved soybean varieties have been developed and disseminated along with production and processing technologies in northeast Nigeria. This has spurned interest in the cultivation of soybean in the Guinea and Sudan savannas of the region (Kamara et al., 2022). Prior to 2004, very little soybean was grown in northeast Nigeria because of a lack of information on appropriate varieties and markets to sell the grains (Amaza et al., 2007). With the intervention of several development projects in the region, soybean is gradually becoming a major grain crop for smallholder farmers. Kamsang (2021) has reported an 86% adoption of soybean cultivation by smallholder farmers in Borno State, northeast Nigeria while Kamara et al. (2022) reported a 75% adoption of improved soybean varieties in the zone by smallholder farmers.

While Nigeria is the second-largest soybean producer in Africa after South Africa (FAOSTAT, 2021), the yield is, on average less than one ton ha^−1^, which is below the potential yield of over 3 tons ha^−1^ (Ronner et al., 2016). Production constraints, such as pests and diseases, drought, poor soil fertility, high pod shattering, and poor agronomic practices, contribute to low soybean yields (Kamara et al., 2014, Khojely et al., 2018). Among the climatic limitations, the occurrence of drought and high temperatures during flowering and grain-filling stages particularly negatively affects the yield of soybean (Bebeley et al., 2022). In the Nigeria savannas, delays in the onset of the rainy season are becoming common (Tofa et al., 2020). Also, long dry spells at the beginning, mid and end of the rainy season are becoming more frequent even in the wetter southern and northern Guinea savannas (Adnan et al., 2017). The combination of late-onset and early cessation of rain has resulted in a shorter rainy season in the savannas from 1971 to 2000 compared to the period from 1941 to 1970 (NIMET, 2008). As a result of the uncertainties in rainfall patterns, rainfed crop production is becoming more variable, and farmers are faced with more risks during production in the savannas of northeast Nigeria. The variability in the onset and end of the rainy season requires knowledge of the best sowing window as a crop management practice for grain crops in the northeast Nigeria savannas.

The soils in the savannas of northeast Nigeria are not only poor in nutrients but are also very heterogenous (Kwari et al., 2011). The variability is induced either by management practices or as a result of differences in texture (Kwari et al., 2011). Variability in soil characteristics may result in variable crop response and performance. For example, variation in soil depth affects the rooting characteristics of crops, with shallow soils restricting root penetration, resulting in contrasting yield responses to nutrients and moisture (Roobroeck et al., 2021). Variations in soil texture, pH, nutrients, organic matter content and slopes are also reported to limit the efficiency of crop response to fertilizer (Palm et al., 2007, Zingore et al., 2007). The wide variability in the climate and soils in northeast Nigeria may therefore influence the performance of soybean across the zone.

While soybean is considered an important crop in the northeast region of Nigeria, there is little information on the performance of this crop across the region. Most of the reported yields from studies conducted in this region have come from a few experimental studies covering very few areas. For example, Kamara et al. (2008) reported a yield of 2610 kg ha^−1^ with a phosphorus application rate of 40 kg P_2_O_5_ ha^−1^. A new industrial crop such as soybean can be valuable to the farmers in northeast Nigeria, but widespread adoption of the crop in areas like northeast Nigeria where the crop is relatively new faces a number of barriers. These include both a lack of reliable information regarding the yield potential of the crop in the diverse production environments, making it difficult to assess the economic value of the crop, and a lack of knowledge regarding appropriate agronomic management such as sowing dates, making crop production risky. Soybean crop management for northeast Nigeria has been based on the management for current production areas in north-central and northwest Nigeria, which are the main soybean growing zones, using, for example, sowing dates, plant density and varieties. This will limit potential yield in the new zones in northeast Nigeria. Crop management needs to be adjusted for different environmental conditions to reduce risks associated with climate and production costs (Battisti et al., 2020), and to increase crop resilience (Halsnaes and Taerup, 2009). Some crop management practices that can be used in new environments to improve yield and crop resilience, include sowing dates (Hu and Wiatrak, 2012, Spehar et al., 2015), and maturity group or varieties (Battisti et al., 2018, Teixeira et al., 2019).

Crop production in northeast Nigeria encompasses a range of climates, soil types, and latitudes, which may cause significant variation in crop performance. The zone has diverse ecologies comprising of the southern and northern Guinea, and the Sudan savannas with significant variations in climate and soil characteristics. There are insufficient resources to conduct extensive field-based research and development activities needed to identify the yield potential of soybean varieties in these diverse environments. The variability in soils, microclimates and socio-economic conditions within the savannas of northeast Nigeria requires the development and deployment of decision support tools that can help in the diagnoses and analysis of problems and opportunities in soybean production under smallholder systems. The use of such tools could assist decision-making at various stages including site selection, evaluation of various management options, selection of crop varieties, and extrapolation and scaling-out of results obtained from a limited area to other areas. Properly calibrated and validated crop models, therefore, provide a valuable tool for assessing the yield potential of improved soybean varieties and for exploring agronomic management strategies to optimize production in the zone.

The Cropping System Model (CSM) CROPGRO-Soybean in the suite of the Decision Support System for Agrotechnology Transfer (DSSAT) (Hoogenboom et al., 2019) is a modelling framework that simulates vegetative and reproductive development, growth and yield of soybean as a function of crop characteristics, weather and soil conditions, and crop management scenarios (Jones et al., 2003). The CROPGRO-Soybean has been used to accurately simulate the growth and yield of soybean for a range of crop management practices in several locations. These include the investigation of variety and sowing time technologies in Brazil (Sampaio et al., 2021), Nigeria (Bebeley et al., 2022), and Thailand (Banterng et al., 2010) and varietal performance of soybean in diverse ecologies in Kenya (Nyambane et al., 2012). There is a need to determine the performance in northeast Nigeria of soybean varieties released for use in other parts of the Nigeria savannas. This study had two objectives: (1) to calibrate and evaluate the performance of the CSM-CROPGRO-Soybean model for simulating growth, development, and yield accurately of diverse soybean varieties under local conditions and (2) to apply the model to investigate the yield potential of the soybean varieties in northeast Nigeria under different sowing windows.

## 2 Materials and methods

### 2.1 CSM-CROPGRO-soybean model calibration and evaluation

**Fig. 1:**
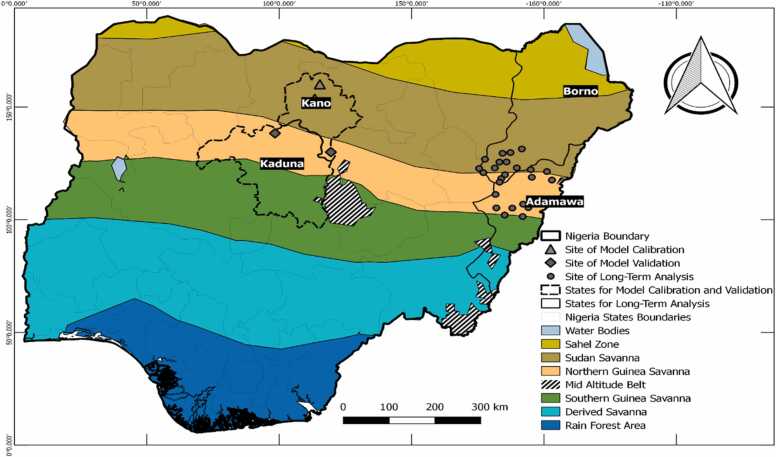
Map of the study area showing agroecological zones boundaries and sites of model simulations.

CROPGRO-Soybean, one of the models running under DSSAT, is a generic physiological process-oriented legume crop model (Kumar et al., 2006)*.* The CSM-CROPGRO-Soybean model within the DSSAT suite simulates plant growth and development from sowing to maturity using a daily time step and ultimately predicts yield (Jones et al., 2003, Banterng et al., 2010, Hoogenboom et al., 2019). The model accounts for vegetative and reproductive developments, photosynthesis, respiration and partitioning, transpiration, root water uptake, soil evaporation, soil water flow, infiltration and drainage (Kumar et al., 2006). The soil, weather, and crop management information are the minimum data sets required to run the model. The simulated physiological processes characterize the crop’s response to the major weather factors, such as temperature, rainfall and solar radiation, and soil characterizations, such as the amount of extractable soil water and nutrients (Banterng et al., 2010). The CROPGRO-Soybean model includes detailed soil and plant nitrogen balance components which simulate nitrogen uptake, nitrogen fixation and nitrogen mobilisation (Hoogenboom et al., 1990). For the calibration of the model, ten experiments were conducted across two sites; Bayero University, Kano (BUK) and Audu Bako College of Agriculture (ABCOA), Dambatta, in the Sudan savanna agroecological zone of Nigeria (Fig. 1). Each experiment consisted of two soybean (TGX 1951–3 F and TGX 1448–2E) varieties that were established in a randomized complete block design (RCBD) and replicated three times from 2016 to 2019. The experiments were conducted under optimum management practices to avoid stresses from water, nutrients, pests and diseases. Parameters measured include days to 50% flowering, days to maturity, grain yield (kg ha^–1^) and total dry matter (kg ha^–1^).

The soybean model was calibrated by determining the cultivar coefficients of the two varieties (TGX 1951–3 F and TGX 1448–2E) which are widely disseminated in northeast Nigeria. Except for variety TGX 1448–2E, the calibration results for TGX 1951–3 F has been reported by Bebeley et al. (2022). The cultivar parameters calibrated and used in DSSAT are shown in Table 1a. The Soil Root Growth Factors for the two sites from soil file that were used during the calibration exercise are given in Table 1b. The CSM-CROPGRO-Soybean model requires 15 cultivar coefficients (CSDL, PPSEN, EM-FL, FL-SH, FL-SD, SD-PM, FL-LF, LFMAX, SLAVR, SIZLF, XFRT, WTPSD, SFDUR, SDPDV, and PODUR) that describe the growth and development characteristics for each individual cultivar. As these were not available for the cultivars used in these experiments, the existing cultivar Jupiter 10 was used as a template at the start of the calibration because it represents the characteristics of tropical soybean varieties. The cultivar coefficients for each cultivar were determined through trial and error of the model and by comparing simulated and observed data, following the procedures described by Hoogenboom et al. (1999). In the CROPGRO-Soybean model, the Genotype-Specific Parameters (GSPs) were calibrated by comparing simulated and measured data for days to 50% flowering, days to physiological maturity, grain yield and total dry matter from the calibration experiments.

**Table 1a:** Calibrated soybean cultivar parameters.

Coefficient	Definition	Unit	TGX1951–3 F	TGX1448–2E
CSDL	Critical Short-day length below which reproductive development progresses with no day-length effect (for short-day plants)	hour	11.37	12.27
PPSEN	Slope of the relative response of development to photoperiod with time (positive for short-day plants)	1/hour	0.340	0.311
EM-FL	Time between plant emergence and flower appearance (R1)	pd*	27.84	24.52
FL-SH	Time between first flower and first pod (R3)	pd	6.000	7.000
FL-SD	Time between first flower and first seed (R5)	pd	14.35	21.52
SD-PM	Time between first seed (R5) and physiological maturity (R7)	pd	21.35	21.41
FL-LF	Time between first flower (R1) and of leaf expansion	pd	15.00	15.00
LFMAX	Maximum leaf photosynthesis rate at 30 0 C, 350 vpm CO2, and high light	mg CO_2_/m^2^/s	1.016	1.010
SLAVR	Specific leaf area of cultivar under standard growth conditions	cm^2^/g	315.3	374.1
SIZLF	Maximum size of full leaf (three leaflets)	cm^2^	230.6	151.8
WTPSD	Maximum weight per seed	g	0.184	0.188
SFDUR	Seed filling duration for pod cohort at standard growth conditions	pd	18.45	24.03
SDPDV	Average seed per pod under standard growing conditions	#/pod	2.090	2.090
PODUR	Time required for cultivar to reach final pod load under optimal conditions	pd	10.00	10.00

*pd = photothermal days.

**Table 1b:** Root growth factor for calibration of the DSSAT-CROPGRO-Soybean model.

Depth (cm)	Root growth factor (soil file)
**BUK-Kano**	
0–28	1
28–58	0.432
58–120	0.161
120–156	0.063
156–210	0.026
**Dambatta**	
0–22	1
22–49	0.492
49–69	0.307
69–116	0.157
116–190	0.047

The capacity of the model to represent local cropping systems was assessed using four independent data sets from P response trials using three phosphorus levels (0, 20 and 40 kg P_2_O_5_ ha^−1^). The experiments were conducted at Doguwa (in 2016) and Zaria (in 2016, 2017, and 2018) in the northern Guinea savanna agroecological zone of the Nigeria savannas (Fig. 1). A split-plot design with three replications was used for each experiment, with the main plot consisting of the phosphorus fertilizer rates and the subplot treatments being the soybean varieties. Data collected included days to 50% flowering, days to physiological maturity, grain and biomass yield. The detailed climate, field observations, soil, and crop management practices used for model evaluation were previously published by Bebeley et al. (2022). The response of the model was evaluated using three different statistical indicators, including root mean square error (RMSE), normalized root mean square error (RMSE_n_) and index of agreement (d-value). The values of RMSE and d-value indicate the degree of agreement between the predicted values and their corresponding observed values. A low RMSE value is desirable. The d-value is a better indicator of model performance, particularly relative to the 1:1 line, and values closer to 1 indicate better prediction, while a d-value of zero indicates no predictability.

### 2.2 Soil and weather data collection for the calibration and validation trials

Soil profile pits were dug in all the experimental areas for the calibration and validation experiments. The generic horizons of the profiles and soil types were classified using the FAO guidelines (FAO, 2006). The soil samples were analysed according to the procedures of IITA (1989). Daily records of rainfall, temperature (minimum and maximum) and solar radiation for the experimental periods were collected from an automated WatchDog weather station device (2000 Series, Spectrum Technologies, Aurora, IL, USA) located adjacent to the experimental locations. The weather data was inputted into the weatherman utility software in the DSSAT v4.7 where it was checked for errors before use.

### 2.3 Long-term seasonal analysis

#### 2.3.1 Description of the sites for the simulation

Locations within the Guinea and Sudan savannas of northeast Nigeria were selected for the simulations. The locations represent the possible soybean production areas of Adamawa and Borno States and are broadly representatives of the climatic and edaphic conditions within northeast Nigeria (Fig. 1). The simulations were performed across 24 selected sites across the two States for the 2 soybean varieties. The sites represent three agro-ecological zones of the Sudan savanna (SS–12 sites), the northern Guinea savanna (NGS–10 sites) and the southern Guinea (SGS–2 sites) savannas (Fig. 1). The NGS and SGS were combined and hereafter referred to as Guinea savannas (GS). The SS has a long dry season with a mono–modal rainfall pattern and a rainy season running from May–October and characterized by high mean temperature (28–32 °C), short growing season (90–110 days) and low rainfall ranging from 600 to 800 mm (Adnan et al., 2017). The soils are highly weathered and fragile, with low clay content (Shehu et al., 2015). The dominant soil class of the site is Alfisol (Dawaki et al., 2013). The growing period in the GS ranged from 151 to 180 days (Jagtap, 1995) for the NGS to 181–210 days (Ayanlade et al., 2013) for the SGS. Annual rainfall in the GS ranges from 900 to 1000 mm for the NGS and1000–1524 mm for the SGS. Maximum and minimum temperatures are 40 and 28 °C, respectively, in the NGS (Atehnkeng et al., 2008). In the SGS, maximum and minimum temperatures are 28 °C and 22 °C, respectively (Ayanlade et al., 2013, Omotosho et al., 2013). The soils are classified as leached ferruginous tropical soils with high clay content and overlying drift materials (Aminu and Jaiyeoba, 2015) in the NGS. The dominant soil types are Alfisols and Entisols (FAO/UNESCO, 1974). In the SGS, the soils have been identified mainly as Lithosols, Ferralic combisols, Feric acrisols, Oxic haplustalfs and Luvisols (FAO/UNESCO, 1974).

#### 2.3.2 Soil characteristics at the simulation sites

The soil parameters used were obtained from on–site soil characterization using geospatially buffering points at least 20 km radius using ArcMap v 10.4. For soil characterization and soil sampling, profile pits were dug in the 24 selected sites in the two States. The profiles and soil types were classified using the FAO guidelines (FAO, 2006). All laboratory analyses were carried out at the Analytical Services Laboratory of IITA according to the procedure of IITA (1989).

The soils in the Guinea savannas (Table 2) were generally deep reaching up to 200 cm profile depth in 60% of the sites. Tawa had the shallowest depth (127 cm) across the sites in this zone. The pH ranged from 6.2 (“slightly acidic”) in Hushere Zum to 9.7 (“strongly alkaline”) in Yola north. Except in Danayel which had soil surface organic content of 0.22% (“very low”), all the other sites in this zone are within the “low” organic carbon class, with Chikila leading with 0.9% organic carbon content. Surface total nitrogen in the soils varied between “very low” (0.013%) at Daneyel to “low” (0.082%) at Chikila. The P content in the top soil can be classified generally as “low” across the sites with the exception of Mbula Kuli, Kikan Kodomti and Daneyel which have P contents of 32.15, 13.76 and 10.99 mg kg^−1^ respectively. Potassium was deficient in about 58% of the sites. However, very high surface K content was found at Nasarawo Demsa (0.89 cmol(+)/kg), Mbula Kuli (0.47 cmol(+)/kg), Hushere Zum (0.43 cmol(+)/kg^−1^), Gwaskara (0.79 cmol(+)/kg) and Kubo (0.78 cmol(+)/kg). Most of the surface soils across the sites were coarse-textured with sand content of 59% and higher. Textural classification of the sites indicated that 83.3% (10 sites) fell into the sandy loam class. The outstanding two sites Chikila and Gwaskara which have higher clay contents of 66% and 58% were classified as clayey soils. The Soils in the Sudan savanna zone (Table 3) are in general deep; reaching up to 200 cm in about 83% of the study sites. The soils in Kabura (110 cm), and Tum (127 cm) were shallower than those of the other sites. With the exception of Balbaya which had a pH value of 6.1, the pH at the top soil across the sites are slightly alkaline. Organic carbon varied widely across the sites, with highest value (1.07%) obtained for Kabura. Lowest organic carbon content (0.12%) was found in Mathau. Total nitrogen content in the top soil was below the threshold level of 0.1% in 11 out of the 12 sites. Phosphorous was also deficient in all the sites with values ranging from 0.76 mg kg^−1^ at Sakwa Hema to 2.83 mg kg^−1^ at Mathau except at Lakundun which had a value of 13.62 mg kg^−1^. Potassium deficiency was observed in 50% of the sites. The values of K at Briyel, Lakundum, Mathau, Puba Vidau, and Tum were above the critical soil K level of 0.3 cmol(+)/kg^−1^ established for the savanna soils. Textural classification also varied across the sites with about 60% of the sites classified as sandy loam. Another 25% which comprised of Jara Dali, Kabura, and Tum fell under sandy clay textural class. Briyel and Puba Vidau are have high clay contents (Table 3).

**Table 2:** Result of soil physico-chemical analysis for each of the identified horizons in the 12 selected sites in the Guinea savanna zone of northeast Nigeria.

Site	Depth (cm)	Horizon	pH (H_2_0)	OC (%)	N (%)	Meh P (mg/kg)	K (cmol (+) /kg)	Sand (%)	Silt (%)	Clay (%)	Bulk Density (gcm^−3^)
Bila Gusi	0–8	Ap	6.5	0.48	0.020	2.14	0.10	67	15	18	1.54
	8–23	AB	6.5	0.38	0.020	1.03	0.10	80	8	12	1.59
	23–51	B1	6.4	0.22	0.010	2.69	0.20	78	4	18	1.66
	51–122	B2	6.3	0.11	0.010	2.00	0.00	68	10	22	1.62
	122–200	B3	6.0	0.19	0.010	1.03	0.30	76	3	21	1.66
Chikila	0–30	Ap	8.5	0.90	0.082	2.55	1.13	15	19	66	2.18
	30–80	Bt1	8.7	0.85	0.057	1.31	0.78	13	19	68	1.87
	80 − 135	Bt2	8.9	0.73	0.058	2.14	0.49	15	19	66	1.80
	135 − 180	C	9.2	0.40	0.021	3.24	0.25	35	13	52	2.03
Daneyel	0–31	Ap	7.0	0.22	0.010	10.9	0.30	81	7	12	1.76
	31–56	AB	6.8	0.11	0.000	2.83	0.30	80	8	12	1.68
	56–89	B	6.6	0.11	0.000	1.59	0.20	77	7	16	1.81
	89–147	Bt	6.4	0.31	0.000	0.76	0.20	74	10	16	2.09
	147–200	CB	6.5	0.11	0.000	1.17	0.20	80	7	13	2.15
Gwaskara	0–50	Ap	7.1	0.34	0.010	11.50	0.10	72	13	15	1.57
	50–77	AB	7.0	0.73	0.045	31.00	0.34	68	16	16	1.50
	77–113	B1	7.4	0.44	0.032	1.72	0.70	20	24	56	1.32
	113–153	B2	7.4	0.48	0.050	1.45	0.79	18	24	58	1.55
	153–200	B3	7.4	0.60	0.032	1.59	0.25	64	10	26	1.30
HushereZum	0–41	Ap	6.3	0.46	0.030	2.41	0.43	74	8	17	1.93
	41–67	B1	6.4	0.21	0.000	1.86	0.15	68	13	18	1.89
	67–81	BC1	6.2	0.22	0.005	2.14	0.13	80	7	12	1.92
	81–106	2B2	6.3	0.23	0.001	2.00	0.10	74	11	14	1.89
	106–143	2BC2	6.4	0.13	0.000	4.77	0.09	78	9	12	1.87
	143–205	2BC3	6.7	0.11	0.000	1.17	0.07	84	5	11	1.89
Kikan Kodomti	0–22	Ap	7.3	0.66	0.040	13.76	0.20	70	9	20	1.76
	22–51	AB	7.0	0.13	0.006	5.32	0.11	82	5	12	1.76
	51–90	Bt1	7.0	0.02	0.001	3.66	0.07	74	11	14	1.60
	90–99	Bt1	7.2	0.29	0.019	2.55	0.07	48	27	24	1.96
	99–150	Bt2	7.0	0.15	0.007	2.83	0.05	74	9	16	1.95
	150–170	Bt3	7.0	0.22	0.016	2.55	0.08	50	23	26	2.00
	170–200	2B2	7.0	0.23	0.000	3.24	0.05	78	8	13	1.94
Kubo	0–33	Ap	7.3	0.46	0.020	1.31	0.80	64	13	23	1.54
	33–106	AB	6.8	0.98	0.080	6.84	0.40	84	5	11	1.49
	106–152	B1	7.3	0.17	0.010	12.90	0.10	68	15	17	1.59
	152–190	B2	7.1	0.44	0.020	3.24	0.30	84	5	11	1.57
	190–200	B3	7.4	0.10	0.000	7.81	0.10	82	5	13	1.66
Mbula Kuli	0–20	Ap	7.8	0.84	0.057	32.15	0.47	59	23	18	1.76
	20–40	AB	7.6	0.43	0.036	10.02	0.17	45	29	26	1.80
	40–68	B1	7.5	0.15	0.009	5.32	0.08	69	17	14	1.90
	68–125	B2	7.5	0.15	0.000	2.69	0.08	81	7	12	1.71
	125–170	Cw	7.2	0.22	0.000	4.49	0.11	91	1	8	0.88
Nasarawo Demsa	0–24	Ap	8.3	0.66	0.057	3.80	0.89	65	15	20	2.01
	24–52	Bt1	7.8	0.53	0.033	1.03	0.56	55	13	32	2.24
	52–83	Bt2	7.6	0.36	0.025	1.59	0.23	47	17	36	2.37
	83–110	Bt3	8.4	0.26	0.030	1.86	0.20	49	19	32	2.36
	110–180	BCw	8.6	0.18	0.011	1.72	0.22	47	19	34	2.04
Tawa	0–15	Ap	6.7	0.62	0.050	3.38	0.21	75	13	12	1.83
	15–32	BA	6.4	0.43	0.027	0.76	0.14	63	21	16	1.62
	32–73	Bv1	6.5	0.37	0.038	1.03	0.29	39	15	46	1.74
	73–127	Bv2	6.5	0.18	0.021	1.86	0.28	53	11	36	1.50
Woroshi	0–14	Ap	6.4	0.54	0.040	1.17	0.36	65	19	16	2.16
	14–51	Cv1	6.0	0.60	0.060	1.86	0.44	37	9	54	2.11
	51–94	Cv2	5.1	0.31	0.040	0.76	0.44	53	9	38	1.91
Yola north	0–24	Ap	6.5	0.40	0.026	1.86	0.09	77	11	12	2.21
	24–54	Bt1	9.3	0.33	0.022	1.72	0.22	49	15	36	2.15
	54–97	Bt2	9.4	0.15	0.020	3.11	0.24	45	17	38	2.03
	97–126	Bc1	9.6	0.17	0.023	3.38	0.29	45	13	42	1.82
	126–155	Bc2	9.7	0.21	0.026	4.07	0.28	39	17	44	2.04

**Table 3:** Result of soil physico-chemical analysis for each of the identified horizons in the 12 selected sites in the Sudan savanna zone of northeast Nigeria.

Site	Depth (cm)	Horizon	pH (H_2_0)	OC (%)	N (%)	Meh P (mg/kg)	K (cmol (+) /kg)	Sand (%)	Silt (%)	Clay (%)	Bulk Density (gcm^−3^)
Balbaya	0–9	AB	6.1	0.29	0.012	1.03	0.05	83	7	10	1.59
	9–22	AB	6.2	0.13	0.006	1.45	0.04	77	11	12	1.61
	22–33	B1	6.3	0.34	0.013	0.89	0.05	79	9	12	1.59
	33–74	B2	7.2	0.34	0.013	5.18	0.06	63	19	18	1.54
	74–200	B3	6.4	0.10	0.003	0.76	0.08	78	10	12	1.63
Briyel	0–15	Ap	8.4	0.39	0.019	2.69	0.36	18	30	52	1.05
	15–52	AB	7.5	0.27	0.010	1.86	0.06	68	17	15	1.08
	52–120	B1	8.0	0.51	0.027	0.76	0.45	18	26	56	1.01
	120–200	B2	7.5	0.88	0.053	3.24	0.22	19	24	57	1.03
Dulmava	1. - 27	Ap	7.5	0.51	0.060	1.03	0.17	67	15	18	1.82
	27–46	Bt	7.2	0.21	0.020	1.31	0.12	61	17	22	1.95
	46–83	2 A	7.1	0.02	0.000	0.89	0.09	79	5	16	2.07
	83–110	2Bt	7.5	0.29	0.030	1.31	0.18	25	37	38	1.93
	110–126	3 A	7.3	0.01	0.000	1.72	0.11	63	19	18	1.71
	126–201	3Bt	7.3	0.17	0.010	1.59	0.10	25	45	30	1.82
Guyaku	0–19	Ap	6.6	0.35	0.030	2.14	0.22	79	9	12	1.70
	19–42	AB	6.8	0.02	0.000	1.45	0.22	79	9	12	1.86
	42–74	B	7.1	0.19	0.010	1.72	0.31	79	5	16	1.83
	74–93	BC	6.7	0.13	0.000	1.59	0.35	69	9	22	2.10
	93–120	C	6.5	0.13	0.000	2.00	0.17	79	9	12	1.63
Jara Dali	0–8	Ap	7.3	0.27	0.023	2.55	0.19	46	18	36	1.55
	8–46	AB	8.3	0.35	0.028	1.45	0.44	14	28	58	1.59
	46–72	B1	6.6	0.33	0.023	1.72	0.26	50	14	36	1.53
	72–103	B2	7.8	0.24	0.018	1.45	0.15	64	12	24	1.53
	103–120	B3	6.8	0.40	0.015	1.59	0.42	53	11	36	1.54
	120–200	B4	7.0	0.10	0.011	1.31	0.28	76	2	22	1.67
Kabura	0–22	Ap	7.0	1.07	0.103	2.41	0.25	34	24	42	1.36
	22–31	AB	7.6	0.14	0.014	1.45	0.27	72	11	17	1.62
	31–101	B1	7.5	0.14	0.011	2.97	0.93	80	6	14	1.33
Kurbo Gayi	0–10	Ap	7.2	0.32	0.013	1.03	0.12	75	9	16	1.04
	10–54	AB	6.9	0.99	0.067	6.56	0.98	50	26	24	0.91
	54–82	B1	6.3	0.19	0.009	1.86	0.10	58	12	30	0.95
	82–125	B2	7.6	0.41	0.023	0.89	0.08	39	35	26	1.25
	125–200	B3	5.8	0.10	0.003	1.45	0.15	68	10	22	1.05
Lakundum	0–16	Ap	7.3	0.73	0.066	13.62	0.55	72	10	18	1.19
	16–81	AB	8.2	0.16	0.018	0.89	0.77	48	14	38	1.22
	81–132	B1	7.1	0.78	0.063	0.89	0.40	36	38	26	1.40
	132–200	B2	7.5	0.45	0.035	2.41	0.36	26	28	46	1.31
Mathau	0–12	Ap	7.4	0.12	0.005	2.83	0.83	90	0	10	1.23
	12–21	AB	6.8	0.30	0.024	2.00	0.38	24	30	46	1.36
	21–47	B1	6.4	0.22	0.007	2.69	0.19	78	3	18	1.14
	47–94	B2	6.6	0.55	0.045	1.17	0.31	24	32	44	0.83
Puda Vidau	0–10	Ap	8.3	0.40	0.021	0.89	0.62	18	19	63	1.32
	10–80	AB	8.3	0.59	0.042	1.45	0.57	18	25	57	1.30
	80–200	B1	7.0	1.06	0.076	1.17	0.77	18	29	53	1.25
Sakwa Hema	0–15	Ap	7.0	0.52	0.042	0.76	0.14	74	9	17	0.85
	15–49	AB	7.8	1.05	0.101	4.21	0.78	18	25	57	1.10
	49–103	B1	7.6	0.16	0.006	0.89	0.08	84	5	11	1.08
	103–170	B2	6.6	0.70	0.073	1.86	0.08	78	9	13	1.10
Tum	0–12	Ap	7.4	0.19	0.009	1.17	0.60	28	24	48	0.87
	12–36	AB	6.8	0.38	0.020	7.95	0.41	80	8	12	0.92
	36–127	B1	6.0	0.30	0.017	0.76	0.34	74	4	22	1.06
	127–200	B2	7.0	0.56	0.041	14.45	0.10	88	0	12	0.87

#### 2.3.3 Weather condition of the simulation sites

Long–term weather data were sourced and downscaled from gridded Climate Hazards Group InfraRed Precipitation with Station data (CHIRPS) for daily rainfall (Funk et al., 2015) and National Aeronautics and Space Administration (NASA) database for Climatology Resource for Agroclimatology http://power.larc.nasa.gov/ that include minimum and maximum air temperature and solar radiation. CHIRPS produced satellite–based rainfall products with relatively high resolutions (5.5 km) and quasi–global coverage (50 °S– 50 °N) for daily, pentadal, and monthly precipitation. The data/parameters in NASA power are provided on a global grid with a spatial resolution of 0.5° latitude by 0.5° longitude. Thereafter, the two datasets were merged using R scripts which was developed to append CHIRPS and NASA power data together, and convert each location into a format readily ingestible by the DSSAT model for the 33 selected sites.

**Fig. 2:**
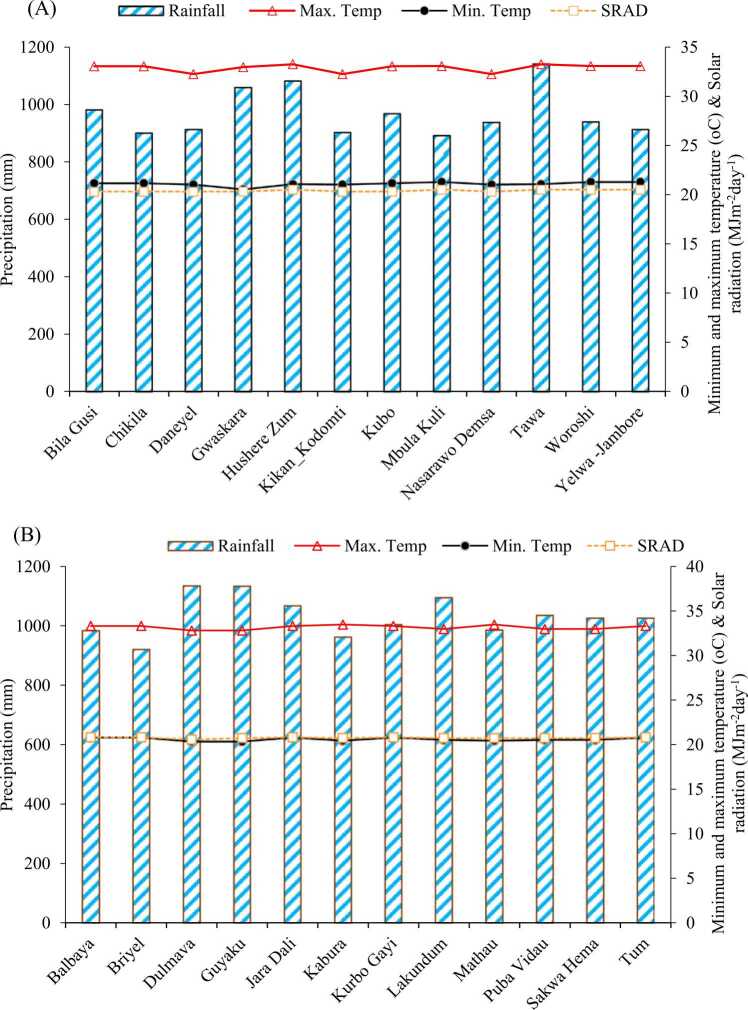
Thirty years (1985–2014) average precipitation, minimum and maximum temperatures of the 12 study sites in Guinea (A) and Sudan (B) savanna agro-ecologies of Nigeria.

Fig. 2 shows the distribution of average precipitation and temperature trends obtained in the study areas over the past 30-year period (1985–2014). The results revealed that the 30-year average precipitation varied among the study sites in both Guinea and Sudan savannas. In the Guinea savanna zone, the average seasonal precipitation over the past 30-year period ranged from 893 to 1143 mm, with Tawa having the highest precipitation while Mbula Kuli recorded the lowest (Fig. 2A). In Sudan savanna, the average seasonal precipitation over the 30-year period ranged from 922 to 1136 mm, with Dulmava having higher precipitation while Briyel recorded the lowest in the Sudan savanna (Fig. 2B). Precipitation was found to be optimum (above 800 mm) for soybean production across all the sites for both agro-ecologies. The air temperature trend also varied among the study sites and the agroecological zones (Fig. 2). In Guinea savanna, average seasonal maximum temperatures ranged between 32.3 and 33.3 °C across the sites over the climatic period, while average seasonal minimum temperatures ranged between 20.6 and 21.3 °C (Fig. 2A). Similarly, in Sudan, the average seasonal maximum temperature across the sites over the climatic period ranged between 32.8 and 33.5 °C while the average seasonal minimum temperature ranged from 20.4 to 20.8 °C (Fig. 2B). Annual solar radiation was found to be slightly higher in Sudan savanna region, with an average of 20.8 and 20.4 MJ m-^2^ day^−1^ for Guinea and Sudan, respectively (Fig. X A, B).

#### 2.3.4 Simulation of soybean performance under different sowing window scenarios in northeast Nigeria

Seasonal analysis was carried out under rainfed–conditions to assess the yield performance of the two soybean varieties at 12 representative sites in each agroecological zone in northeast Nigeria under varying sowing windows. The simulation was applied over a 30–year period (1985–2014) at varying sowing windows using the daily rainfall, temperature and solar radiation and soil information obtained from the three locations. The model was set to consider four sowing windows (SWs) implemented in CROPGRO–Soybean Model: June 15–25 (SW1), June 26–July 5 (SW2), July 6–16 (SW3), July 17–27 (SW4). Sowing was carried out based on the treatments with conditions set to sow when a total rainfall is above 10 mm within the last three days before sowing in each simulation year. Generally, the sowing density was 53.3 plants m^–2^, sown at a soil depth of 4 cm. The model was set to supply 40 kg P_2_O_5_ ha^–1^ at sowing using TSP fertilizer material. The model was set to harvest at harvest maturity. The standard deviations, the mean, maximum and minimum yields for 30 years were calculated for each variety and location. The level of variability (high or low percentage) determined whether the variety is adapted to the site based on mean grain yield of ≥ 1500 kg ha^–1^ as threshold.

## 3 Results

### 3.1 Model calibration and validation

**Fig. 3:**
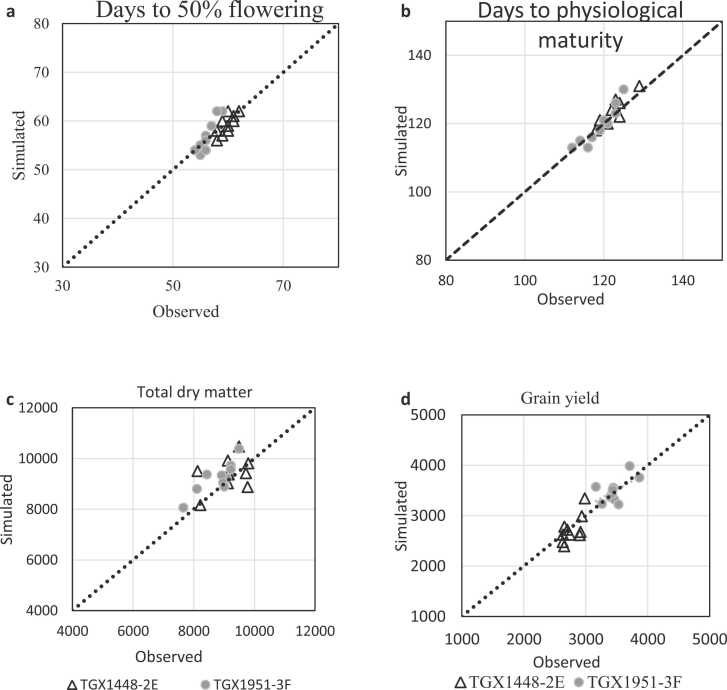
(a)–Observed vs. simulated days to 50% flowering using calibration experiment conducted 2016–2019 growing seasons for variety TGX 1448–2E (RMSE = 1.3 days, RMSE_n_ =2.3%, D= 0.8); TGX1951–3 F (RMSE =1.95 days, RMSE_n_ =3.5%, D=0.83). Fig. 3b – Observed vs. simulated days to physiological maturity using calibration experiment conducted 2016–2019 growing seasons for variety TGX 1448–2E (RMSE = 2.0 days, RMSE_n_ =1.7%, D= 0.92); TGX1951–3 F (RMSE =2.21days, RMSE_n_ =1.9%, D=0.94). Fig. 3c – Observed vs. simulated total dry matter using calibration experiment conducted 2016–2019 growing seasons for variety TGX 1448–2E (RMSE = 673.75 kgha^−1^, RMSE_n_ =7.3%, D= 0.63); TGX1951–3 F (RMSE =538.46 kgha^−1^,RMSE_n_ =6.1%, D=0.8). Fig. 3d – Observed vs. simulated grain yield using calibration experiment conducted 2016–2019 growing seasons for variety TGX 1448–2E (RMSE = 198.45 kgha^−1^, RMSE_n_ =7.1%, D= 0.74); TGX1951–3 F (RMSE =197.7 kgha^−1^,RMSE_n_ =5.7%, D=0.75).

**Fig. 4:**
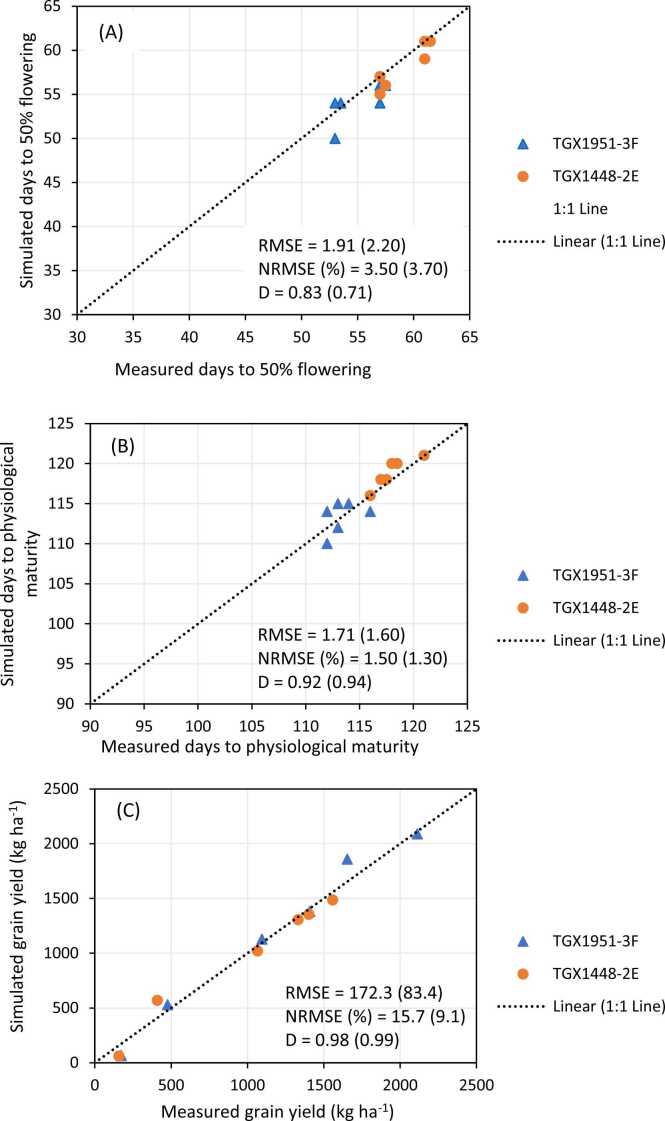
– Observed vs. simulated days to flowering (A), days to physiological maturity (B) and grain yield (C) for variety TGX 1448–2E and TGX1951–3 F using validation experiment conducted at Doguwa and Zaria. Values in brackets are the statistical indices for variety TGX 1448–2E.

The CROPGRO-Soybean model calibration adequately simulated days to 50% flowering, days to 95% physiological maturity, total dry matter and grain yield of the two varieties using the calculated coefficients (Figs. 3(a-3d)). For the variety TGX 1448–2E, the d-index values were 0.80, 0.92, 0.63 and 0.74 with corresponding RMSE values of 1.3 days, 2 days, 674 kg ha^−1^ and 198 kg ha^−1^ for days to flowering, days to maturity, dry matter and grain yield, respectively. For TGX 1951–3 F the d-index values were 0.83, 0.94, 0.80 and 0.75 with corresponding RMSE values of 1.95 days, 2.2 days, 539 kg ha^−1^ and 197 kg ha^−1^ for flowering, maturity, dry matter and grain yield, respectively. Generally, the calibration results showed high prediction accuracy with RMSEn < 10% for all the measured variables. The accuracy of the CROPGRO-Soybean model simulations and performance of genetic coefficients were assessed by comparing the simulated values with independent data sets collected from field experiment (Section 2.1). The model's evaluation of all measured parameters was good with RMSEn < 4% for phenology, < 10% for grain yield of TGX 1448–2E and < 20% for grain yield of TGX 1951–3 F in both locations (Fig. 4). The values of RMSE were 1.9 and 2.2 days for flowering, 1.6 and 1.7 days for physiological maturity and 84 and 172 kg ha^−1^ for grain yield each for TGX 1951–3 F and TGX 1448–2E, respectively. In all cases, d-index values for all the parameters measured were above 0.70 indicating that the model is robust and accurate in measuring phenology and grain yield.

#### 3.1.1 Long term seasonal analysis

**Fig. 5:**
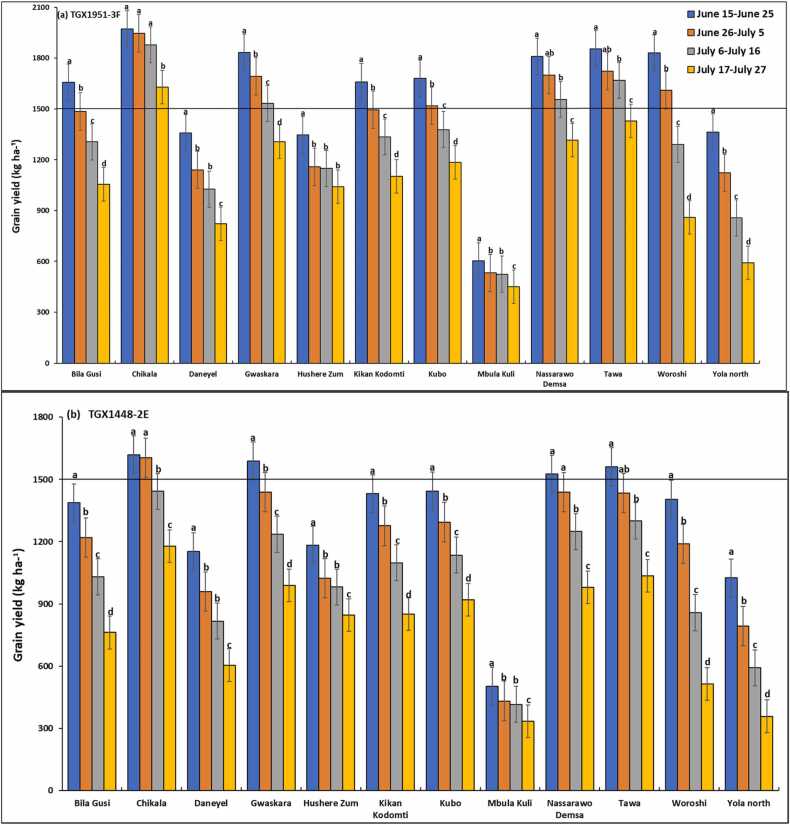
Mean yield for different sowing windows predicted by the CROPGRO-Soybean model for soybean varieties (a) TGX1951–3 F and (b) TGX 1448–2E in the Guinea savannas of northeast Nigeria. Error bars show standard deviation of model estimates across 30 seasons. Different letters for bars indicate significant difference within location at 5% probability.

**Fig. 6:**
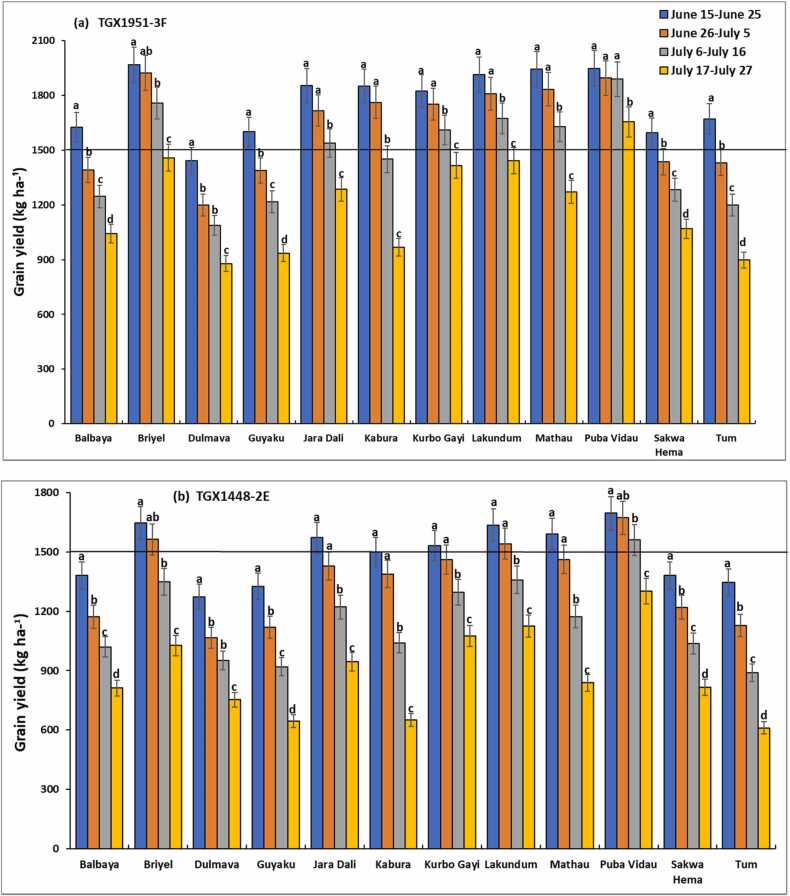
Mean yield for different sowing windows predicted by the CROPGRO-Soybean model for soybean varieties (a) TGX1951–3 F and (b) TGX 1448–2E in the Sudan savanna of northeast Nigeria. Error bars show standard deviation of model estimates across 30 seasons. Different letters for bars indicate significant difference within location at 5% probability.

Results of the 30-year simulation of the rainfed soybean yield vary considerably among sites within agroecological zones and among sites between agroecological zones (Fig. 5, Fig. 6). The yield was higher in the Sudan savanna zone than in the Guinea savanna zone (Fig. 5, Fig. 6). Across sowing windows, the simulated yield was similar for Chikala in the GS (Fig. 5) and Puba Vidau in the SS (Fig. 6), which were higher than the yield simulated for the other locations. The least yield was simulated for Mbula Kuli in the southern part of the Guinea savanna zone (Fig. 5). Simulated grain yield was generally higher for the variety TGX 1951–3 F than for that of TGX 1448–2E in all the sites across agroecological zones.

In addition to location and soybean variety, the yield was also dependent on sowing window. Mean simulated grain yield of the two varieties decreased with delay in sowing in the two agroecological zones. In the Guinea savannas, optimum sowing windows varied between the two varieties. Using grain yield of ≥ 1500 kg ha^–1^ as a threshold, the simulated optimum sowing window for TGX 1951–3 F was June 15–25 in two of the sites (Bila Gusi, Kikan Kodomti), beyond which the yield declined by 10–46% (Fig. 5a). The optimum sowing window in Kubo and Woroshi was June 15–July 5 with yield declining by 12–75% if sown beyond this window. The most suitable sowing window at Gwaskara, Nasarawo Demsa and Tawa, were Jun 15–July 16, with mean grain yield above the threshold of ≥ 1500 kg ha^–1^ (Fig. 5a). The most suitable sowing window at Chikala was June 15–July 27 with mean yield ranging from 1629 to 1971 kg ha^–1^. Sowing within sowing windows of June 15–June 25, June 26–July 5 and July 6–July 16 would produce a similar yield. The performance of TGX1951–3 F was generally below optimum across all sowing windows in Mbula Kuli (451–602 kg ha^–1^), Daneyel (822–1358 kg ha^–1^), Hushere Zum (1042–1346 kg ha^–1^), and Yola north (592–1364 kg ha^–1^). Low yield was generally simulated for TGX 1448–2E for most of the sites in the Guinea savanna except at Gwaskara, Nasarawo Demsa, Tawa and Chikala (Fig. 5b). Except for Chikala, the optimum sowing window for TGX 1448–2E in these sites was simulated for June 15–June 25 with mean yield ranging from 1525 to 1588 kg ha^–1^. The suitable sowing window for Chikala was June 15–July 5 with simulated grain yield ranging from 1605 to 1619 kg ha^–1^.

In the Sudan savanna zone, the most suitable window for sowing TGX 1951–3 F at Guyaku, Balbaya, Sakwa Hema, and Tum was June 15–25, with grain yield ranging from 1597 to 1672 kg ha^–1^ (Fig. 6a). The optimum sowing window at Kabura was simulated for June 15–July 5 with mean yield ranging from 1762 to 1850 kg ha^–1^. Simulated yield decline by 18–45% with sowing beyond July 5. The optimum sowing window was June 15–July 16 for five sites (Briyel, Lakundum, Jara Dali, Kurbo Gayi and Mathau) with grain yield, ranging from 1538 in Jara Dali to 1967 kg ha^–1^ in Briyel. Sowing beyond July 16 significantly reduced grain yield by 15–22%. The result shows that Puba Vidau has the widest suitable sowing window of between June 15–July 27, with grain yield ranging from 1588 to 2002 kg ha^–1^. Poor yield was generally simulated for TGX 1448–2E for six of the sites in the Sudan savanna zone (Fig. 6b). The most suitable sowing window is June 15–June 25 at Jara Dali, Kabura, Kurba Gayi, and Mathau, with mean yield ranging from 1500 to 1591 kg ha^–1^. Sowing beyond this window reduces yield below the threshold of ≥ 1500 kg ha^–1^. Optimum yield was obtained with sowing on June 15–July 5 at Briyel and Lakundum with a yield range of 1562–1681 kg ha^–1^ and June 15–July 16 at Puba Vidau with yield ranging from 1560 to 1695 kg ha^–1^.

**Fig. 7:**
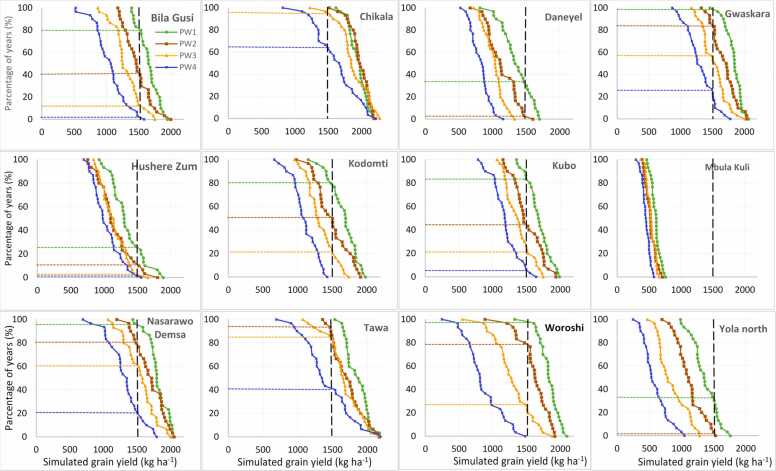
Cumulative probability distributions of simulated soybean (TGX1951–3 F) yield under four different sowing windows (PW1 =June 15–25, PW2 =June 26-July5, PW3 =July 6–16, PW4 =July 17–27) for twelve diverse locations in the Guinea savanna of northeast Nigeria.

**Fig. 8:**
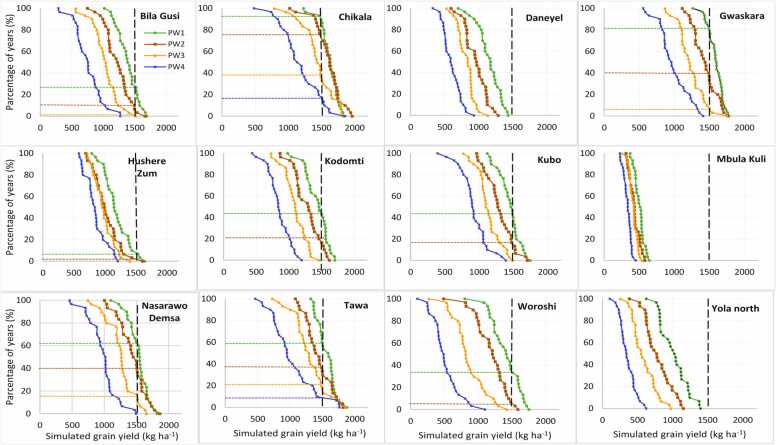
Cumulative probability distributions of simulated soybean (TGX 1448–2E) yield under four different sowing windows (PW1 =June 15–25, PW2 =June 26-July5, PW3 =July 6–16, PW4 =July 17–27) for twelve diverse locations in the Guinea savanna of northeast Nigeria.

**Fig. 9:**
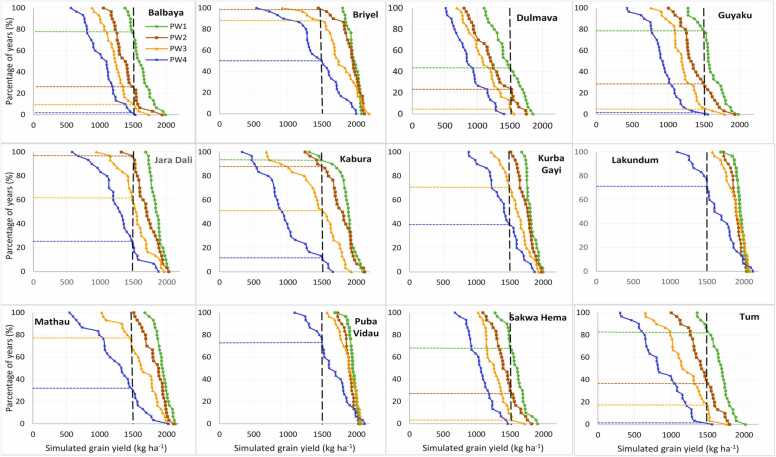
Cumulative probability distributions of simulated soybean (TGX1951–3 F) yield under four different sowing windows (PW1 =June 15–25, PW2 =June 26-July5, PW3 =July 6–16, PW4 =July 17–27) for twelve diverse locations in the Sudan savanna of northeast Nigeria.

**Fig. 10:**
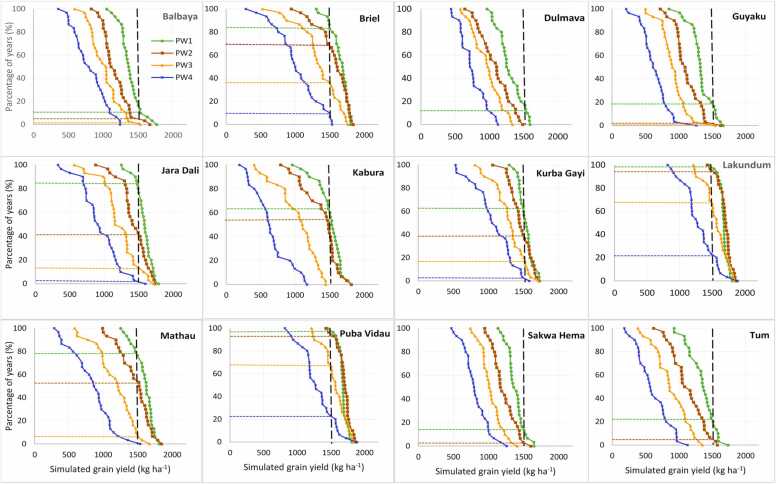
Cumulative probability distributions of simulated soybean (TGX 1448–2E) yield under four different sowing windows (PW1 =June 15–25, PW2 =June 26-July5, PW3 =July 6–16, PW4 =July 17–27) for twelve diverse locations in the Sudan savanna of northeast Nigeria.

Soybean production risk was assessed across the target sites in the region through the cumulative probability distribution graphs (Fig. 7, Fig. 8, Fig. 9, Fig. 10) created by ordering the simulated yield from smallest to largest. The yield threshold that must be met or exceeded for profitable soybean production in the region was set at 1500 kg ha^–1^. In the Guinea savanna zones, the probability of achieving at least ≥ 1500 kg ha^–1^ with TGX1951–3 F is 80% with sowing on June 15 – June 25 in Bila Gusi, Kikan Kodomti, and Kurbo Gayi, beyond which the probability will drop to less than 50% (Fig. 7). The desired grain yield of ≥ 1500 kg ha^–1^ will be achieved in Gwasakara, Nasarawo Demsa, and Woroshi in 80–100% of the years if sown between June 15 and July 5. Sowing beyond July 5, would reduce the probability to between 20% and 65%. The probability of achieving at least ≥ 1500 kg ha^–1^ is 85–100% with sowing between June 15 and July 16 at Chikala and Tawa. Sowing beyond July 16 would reduce the probability of achieving the desired yield to between 20% and 60% of the years. In Daneyel, Hushere Zum, Mbula Kuli and Yola north, the probability of achieving a yield of at least ≥ 1500 kg ha^–1^ is between 0% and 30% of all the years, irrespective of sowing window (Fig. 7). For TGX1448–2E, the probability of achieving the desired yield of ≥ 1500 kg ha^–1^ is 85% at Chikala, 80% at Gwaskara, 65% at Nasarawa Demsa and 60% at Tawa when sowing between June 15 and June 25 (Fig. 8). This probability reduced to 75% at Chikala with delay in sowing to between June 26 and July 5. With sowing beyond June 25, the probability of achieving the desired yield is 5–40% in Gwaskara, 15–40% in Narasaro Demsa, and 25–38% in Tawa. In 8 of the locations, there is very low probability (0–45%) of achieving the desired yield across all sowing windows for this variety.

At Briyel, Lakudum, Mathau, and Puba Vidau in the Sudan savanna agroecological zone, the yield threshold of ≥ 1500 kg ha^–1^ for TGX1951–3 F is achieved in 80–100% of the years with sowing between June 15 and July 16 beyond which the chances of achieving this yield threshold will reduce to 38–70% (Fig. 9). At Balbaya, Guyaku, and Tum, the probability of achieving the desirable threshold is 80% with sowing from June 15–25 beyond which the chances of achieving this threshold will reduce to 30–40%. At Jara Dali, Kabura, and Kurba Gayi, the desired yield is achieved in 85–100% of the years with sowing between June 15 and July 5 beyond which the probability reduces to 40–60%. The probability of achieving the desired yield among all the sowing windows is less than 70% at Sakwa Hema. The chances of achieving the desired yield are very low in most of the locations in the Sudan savannas for TGX1448–2E (Fig. 10) except in Briyel (80–85% for sowing between June 15 and July 5), Jara Dali (85% for sowing on June 15–25), Lakundum (90–95% for sowing between June 15 and July 5), Mathau (79% for sowing on June 15–25 sowing window) and Puba Vidau (90–95% for sowing between June 15 and July 5).

## 4 Discussion

The results of model calibration showed that the genetic coefficients for the two soybean varieties resulted in simulated growth parameters and yield that were in good agreement with their corresponding observed values, consistent with the findings of Bebeley et al. (2022). In all the locations, there was a good fit in the model prediction of days to 50% flowering, days to physiological maturity and grain yield with low RMSE and high d-index values. These results are also consistent with those obtained in tropical environments in Kenya (Nyambane et al., 2012), and Mozambique (Talacuece et al., 2016), where the model accurately reproduced observed values for growth and grain yield. These results of the model evaluation also showed that the model was able to reasonably simulate the phenology and grain yield of soybean for different P-fertilizer rates with low RMSE and high d-index, which is consistent with reports by Bebeley et al. (2022), which showed that the model accurately reproduced observed values for phenology and grain yield of soybean over a range of P rates in the Nigeria savannas. The results also agree with the findings of Naab et al. (2015), who observed high accuracy of prediction with P-fertilization rates in northern Ghana when simulating for groundnut growth using the CROPGRO-Peanut model. Similarly, Halder et al. (2017) reported that the CROPGRO-Peanut model was able to reasonably simulate pod yield and final biomass with low normalized root mean square error (RMSEn), low absolute root mean square error (RMSEa) and a high coefficient of determination (R2 > 0.7) over a wide range of sowing dates and different phosphorus fertilization levels in eastern India. This result conﬁrms the ability of the CROPGRO-Soybean model to accurately predict growth and yield of rainfed soybean under different management practices in the Nigeria savannas.

The long-term simulations strongly indicate that soybean should have a high mean yield in northeast Nigeria if sowing is properly timed. Similar simulation results were obtained for northwest Nigeria by Bebeley et al. (2022). There is however strong influence of locations between and within climatic zones. While the desired simulated yield was achieved in some locations, low yield was simulated in 7 out of 12 locations in the Guinea savanna agroecological zone and in 5 out of 12 locations in the Sudan savanna agroecological zone for TGX 1951–3 F. Low yield was simulated in all locations for TGX 1448–2E except at Chikala in the Guinea savanna and Puba Vidau in the Sudan savanna zone.

Contrary to earlier findings elsewhere in the Nigeria savannas, (Bebeley et al., 2022 for soybean, Tofa et al., 2020 and Beah et al., 2020 for maize), higher yields were simulated for most of the locations in the Sudan savannas than for the Guinea savannas across the two States This is unexpected because higher crop yields are usually obtained in the Guinea savannas than in the Sudan savannas because of higher rainfall and longer growing season in the Guinea savannas than elsewhere in the savannas. Average rainfall in both zones was however, more than 1000 mm per year, suggesting that rainfall limitation may not be the most important determinant of soybean yield in the two zones. Bhatia et al. (2008) reported that the simulated yield of rainfed soybean increased with increasing rainfall from 420 to 1240 mm in some regions in India. They also reported that the observed marginal increase in actual yield on farmers’ fields in response to increasing rainfall was only up to 700 mm and between 700 and 900 mm of rainfall there was no substantial change in the yield. They also found that an increase in rainfall beyond 900 mm resulted in a negative impact on the actual yield. The negative impact of rainfall beyond 900 mm could be due to poor drainage conditions and resultant water-logging in the farmers’ fields, indicating the need for adoption of management practices to overcome the problem of poor drainage and water-logging. In the case of northeast Nigeria, high rainfall in the Guinea savannas may have caused leaching of nutrients in the largely sandy soils in these zones (Fig. 2a).

The yield response between 400 and 900 mm reported by Bhatia et al. (2008) shows the importance of the factors other than rainfall and water availability which limit the realization of rainfed potential of the crop. Differences in soil type and fertility may have resulted in differences in soybean yield among the 24 sites and between the agroecological zones in northeast Nigeria. Though three of the sites in the GS and one in the SS had P levels above the required level of 7 mg kg^− 1^ soil, all the other sites were poor in P, suggesting that differences in soil P may not be responsible for the differences among the sites for grain yield of the soybean crop. Moreover, a sufficient quantity of P at the rate of 40 kg P ha^−1^ was supplied during the simulation analysis. Yields in Briyel, Lakundum, Jara Dali, Kurbo Gayi, Mathau, and Puba Vidau were significantly higher than that of the other locations, largely due to the high clay and organic matter content of the soils in these locations (Table 2, Table 3). The soils in the Sudan savanna zone are also higher in clay and organic carbon than those of the Guinea savannas leading to high retention of nutrients. Bebeley et al. (2022) also reported lower simulated yield for diverse soybean varieties in the southern Guinea savanna than those of the northern Guinea and Sudan savanna zones of northwest Nigeria despite the higher rainfall in the southern Guinea savanna. They attributed this to the high sand content of the soils and very high rainfall in the southern Guinea savanna zone, which may lead to the leaching of nutrients, and waterlogging. Solar radiation was also higher in the Sudan savanna than in the Guinea savanna. Under non-limiting water conditions, Bhatia et al. (2008) reported that simulated soybean yield was highly correlated with solar radiation in 24 locations in India.

A higher yield was generally simulated for TGX 1951–3 F than for TGX 1448–2E in all the locations. Bebeley et al. (2022) also simulated higher yield for TGX1951–3 F over a range of sowing windows than for other soybean varieties in northwest Nigeria. The higher yield of TGX 1951–3 F is also supported by the observed values in the validation trials where under optimum P application, it produced a yield that was higher than that of TGX 1448–2E (Fig. 4). TGX 1951–3 F is a robust and early-maturing variety which is less photosensitive and adapted to the northern Guinea and Sudan savannas of West Africa, where the rains usually start in late June and end in early October. This variety, therefore, fits well in the savannas of West Africa, where the growing season is becoming shorter because of climate change. In times of early cessation of rainfall, this variety will perform better than the late-maturing TGX 1448–2E. TGX 1448–2E is widely cultivated in northern Nigeria, being one of the non-shattering older varieties developed for the West Africa savannas. It stores well and does not have a problem with germination compared to other older varieties. The low yield simulated for this variety across sowing windows in all the sites may, however, be due to the fact that it is late-maturing and photo-sensitive. If sown earlier, flowering may be delayed because of high photosensitivity and if sown later in June or July, the rains may cease before it flowers and complete the life cycle. It may, therefore, not perform well in the zones where the length of the growing season is shorter.

For TGX 1951–3 F, yield was above the threshold of 1500 kg ha^−1^ in 4 (Gwaskara, Nasarawo Demsa, Tawa, and Chikala) out of 12 sites in the GS and 7 (Briyel, Lakundum, Puba Vidau, Jara Dali, Kabura, Kurbo Gayi, and Mathau) out of 12 sites in the SS. Chikala, Briyel, Lakundum, and Puba Vidau were the most productive sites for this variety. This may be due to the deeper soil, and high clay and organic matter contents in these sites coupled with relatively good rainfall for this early-maturing variety. Results show that, though TGX 1448–2E is widely cultivated as one of the early introductions across the Nigeria savannas (Kamara et al., 2009), it should not be recommended for cultivation in northeast Nigeria or if recommended, it should be sown earlier than June 15 provided the rains establish earlier and there is sufficient moisture. Soybean yield was also influenced by sowing windows.

Simulation results show that the optimal sowing windows depend on the location and soybean variety. The location and varietal effects are in turn influenced by soil types, rainfall amount, and pattern across sites and AEZs (Akinseye et al., 2020). For TGX 1951–3 F, sowing window at Bila Gusi and Kikan Kodomti in the GS is short, lasting only 10 days (June 15–25). The probability of achieving the desired yield of TGX 1448–2E is low for any sowing window in those two locations suggesting that it is risky to cultivate these two varieties in those locations. The length of sowing period for TGX 1951–3 F in Woroshi is 20 days (June 15-July 5), 31 days in Gwaskara, Nasarawo Demsa and Tawa, (Jun15-July 16) and 41 days in Chikala (June 15-July 26) which gives ample time for land preparation and sowing. The result also suggests that sowing can be delayed to third week of July with a significantly low risk of crop failure. Generally, TGX 1448–2E should be sown in the June 15–25 window for all locations if no other suitable variety is available except in Chikala where sowing can be delayed to July 5. Results show that Mbula Kuli, Daneyel, Hushere Zum, and Yola north are not suitable for cultivation of the two varieties probably due to the high sand content of soils in these locations. In the Sudan savanna, zone, the length of sowing window for TGX 1951–3 F is 10 days (June 15–25) at Guyaku, Balbaya, Sakwa Hema, and Tum, 20 days at Kabura (June 15-July 5) beyond which simulated yield will decline by 18–45% with significant low probability of achieving the desired yield. Results show that the desired yield will be achieved at 6 of the sites (Briyel, Lakundum, Jara Dali, Kurbo Gayi, Mathau, and Puba Vidau) with a significantly low risk of crop failure for all sowing windows for which yield was simulated. In a similar study by Bebeley et al. (2022) in northwest Nigeria, the simulated yields of the medium-maturing variety TGX1904–6 F declined by 12–70% and the early-maturing TGX 1951–3 F by 10–66% when sown beyond June 28 in the Sudan savanna zone. In northern Guinea savanna zone, the predicted yield decline by 10–33% for TGX 1904–6 F and 8–37% for TGX 1951–3 F when sown beyond July 12. The probability distributions can be used by farmers as risk assessment tool as they contemplate growing soybean in the northeast Nigeria. For decision to grow soybean and achieve at least the desired yield of 1500 kg ha^–1^, the appropriate panel of Fig. 7, Fig. 8, Fig. 9, Fig. 10 can be used to determine the probability of obtaining at least that yield at that location with the given sowing window. Where the probability of achieving the desired yield of 1500 kg ha^−1^ is quite low at any sowing window, farmers do not have to grow the crop.

While sowing date and soybean maturity period were found to significantly influence soybean yield in northeast Nigeria, other crop management practices which equally influence soybean yield were not considered in the simulations. Other crop management practices that can be used in new environments to improve yield and crop resilience include irrigation (Justino et al., 2019, Battisti et al., 2020) and plant density (Sampaio et al., 2021). While rainfall was a major factor for soybean production in the region, plant density could influence soybean growth in addition to the soil texture. In this study, soybean plant density was set at 53.3 plants m^−2^ for all varieties and sites. This blanket density may not be needed for all the varieties and locations, given the high cost of seeds. The results of seasonal analysis over 33 seasons showed that higher plant density (40 plants m^−2^) resulted in higher yield than lower plant density for all locations, sowing dates and maturity groups in Brazil (Sampaio et al., 2021). The authors also found sowing date by planting density interactions significant in these locations. Further research is therefore needed to look at the influence of other crop management practices for soybean in northeast Nigeria.

## 5 Conclusions

In this study, CROPGRO-Soybean model was calibrated, evaluated and applied to assist in providing decision support on the optimum sowing window for soybean in the northeast of Nigeria. The results for model calibration and evaluation showed that simulated growth and yield of soybean were in good agreement with their corresponding observed values. Thus, the CROPGRO-Soybean model can be successfully used to stimulate growth and yield of soybean for major soybean growing regions in Nigeria and the West African savannas at large. The model simulations showed that soybean could be grown in northeast Nigeria. Average grain yield is, however, dependent on site, agroecological zone, variety and sowing window. The simulated yield was higher in the Sudan savanna than in the Guinea savanna agroecological zone despite the long growing period in the later. The simulated grain yield of the early-maturing soybean variety TGX 1951–3 F was higher than that of the late-maturing TGX 1448–2E for all sowing windows. Low yield was generally simulated for TGX 1448–2E for all sowing windows in both agro-ecozones suggesting that it is not suitable for cultivation in northeast Nigeria. For TGX 1951–3 F, yield was above the threshold of 1500 kg ha^−1^ in 5 out of 12 sites in the GS and 7 out of 12 sites in the SS. Results show that Mbula Kuli, Daneyel, Hushere Zum, and Yola North are not suitable for cultivation of the two varieties, probably due to the high sand content of soils in the locations. The optimal sowing windows depend on the location and soybean variety. For TGX 1951–3 F, sowing can be delayed to third week of July at Gwaskara, Nasarawo Demsa, Tawa and Chikala in the GS with low risk of crop failure. In SS, the desired yield will be achieved at Briyel, Lakundum, Jara Dali, Kurbo Gayi, Mathau, and Puba Vidau with a significantly lower risk of crop failure for all sowing windows. While the sowing date and soybean maturity period were found to significantly influence soybean yield in northeast Nigeria in this study, further research is needed to simulate the influence of other crop management practices, such as planting density and supplementary irrigation, on soybean performance in northeast Nigeria.

## Funding

The Bill and Melinda Gates Foundation funded the field studies to obtain climate and field data to calibrate and validate the CROPGRO Model in northern Nigeria through the project Putting Nitrogen Fixation into Use for Smallholder farmers in Africa with Grant no. OPP1020032. Soil data were collected from locations in Borno and Adamawa State and analysed with funds from USAID under the project Integrated Agriculture Activity Agreement No.72062018IO00002.

## CRediT authorship contribution statement

**Alpha Y. Kamara:** Conceptualization, Methodology, Model evaluation, Formal analysis, Resources, Writing – original draft, Writing – review & editing, Funding acquisition. **Jenneh F. Bebeley:** Conceptualization, Methodology, Field experimentation, Data curation, Model evaluation, Formal analysis, Writing – original draft, Writing – review & editing. **Kamaluddin T. Aliyu:** Conceptualization, Methodology, Data curation, Writing – original draft, Writing – review & editing. **Abdullahi I. Tofa:** Conceptualization, Methodology, Field experimentation, Data curation, Model evaluation, Formal analysis, Writing – original draft, Writing – review & editing. **Lucky Omoigui:** Conceptualization, Methodology, Writing – original draft, Writing – review & editing. **Reuben Solomon:** Conceptualization, Methodology, Field experimentation, Data curation, Model evaluation, Formal analysis, Writing – original draft, Writing – review & editing. **Folorunsho M. Akinseye:** Conceptualization, Methodology, Model evaluation, Formal analysis, Writing – review & editing.

## Declaration of Competing Interest

The authors declare the following financial interests/personal relationships which may be considered as potential competing interests: Alpha Yaya Kamara reports financial support was provided by Bill and Melinda Gates Foundation.

## Data Availability

Data will be made available on request.
